# The conditions for women's autonomy: Statistical data for Valle del Cauca

**DOI:** 10.1016/j.dib.2020.105751

**Published:** 2020-05-21

**Authors:** Lina Buchely, Heidy Acevedo, Salome Arias-Arevalo, Edgar Benítez, Carolina Borda Niño, Erika Márquez, Juan Milanese, Enrique Rodríguez, Viviam Unás, Blanca Zuluaga

**Affiliations:** a Universidad Icesi, Director of the Observatorio para la Equidad de las Mujeres; b Universidad Icesi, Coordinator of the Observatorio para la Equidad de las Mujeres; c Universidad Icesi, Research Assistant of the Observatorio para la Equidad de las Mujeres and student of the Master's Degree in Social and Political Studies; d Universidad Icesi, Social Sciences Department, Cali, Colombia; e Fundación WWB Colombia, Chief research officer, Fundación WWB Colombia and visiting lecturer, Sociology and Gender Studies Department, FLACSO Ecuador; f Universidad Icesi, Director of the Sociology Program; g Universidad Icesi, Head of the Department of Political Studies; h Universidad Icesi, Director of the Center for interdisciplinary, legal, social and humanistic studies (CIES); i Universidad Icesi, Head of the Pedagogy Department, Cali, Colombia; j Universidad Icesi, Economics Department, Cali, Colombia

**Keywords:** Autonomy, women, gender equality, stratified sampling, Colombia

## Abstract

In this article, we describe the data set on theconditions for women's autonomy in the municipalities of Cali, Buenaventura, Yumbo, and Jamundí (Colombia). The database was developed by the Observatoriopara la Equidad de las Mujeres (OEM), an entity resulting from an alliancebetween Universidad Icesi and Fundación WWB Colombia. The OEM's purpose is toconstruct measurements that make it possible to account for women's autonomy,based on three main dimensions: physical autonomy (associated with aspects ofpersonal and family life), economic and financial autonomy (the type of work,income, decision-making with respect to these aspects, entrepreneurship orbusiness units, among others), and leadership and public participation(electoral practices, type of participation, relation to institutions, etc.),while fundamentally, considering women's capacity to make decisions onthese  aspects, based on both theirperception and their actions. As well as helping to overcome the limitations ofexisting information, this primary data collection is aimed at generatingvalid, reliable and high-quality inputs to formulate local and national publicpolicies to improve the living conditions of women in Colombia's Pacificregion. The survey, designed and applied by the OEM, was first implemented inOctober and November 2018 through multi-stage probability sampling. The samplesize was 1,507 women in the four mentioned municipalities.

Specifications Table

**Table:** 

Subject	Gender studies
Specific subject area:	Women's autonomy
Type of data:	Text, ordinal, nominal and interval-ratio variables
How data were acquired:	Through personal surveys
Data format:	Unprocessed (cleaned)
Parameters for data collection:	Multi-stage probabilistic sampling
Description of data collection:	The sample was applied in four municipalities of Valle de Cauca: 1) Cali; 2) Jamundí; 3) Buenaventura and 4), Yumbo. We implemented a multi-stage probability sampling method to select units by simple random sampling. The sample was stratified by political division and socioeconomic strata. The political division was by Urban Planning Units (UPU) in the case of Cali, and by comunas for Yumbo, Jamundi and Buenaventura.
Data source location:	Cali, Buenaventura, Yumbo y Jamundí- Valle del Cauca, Colombia Cali: 3°27′00″N 76°32′00″O Buenaventura: latitude 3,5833299 and longitude -77, in the northern hemisphere. Yumbo: latitude 3.58234 and longitude -76.4914627, in the northern hemisphere Jamundí: Longitude: O76 ° 32′12.98 "Latitude: N3 ° 15′38.99"
Data accessibility	Repository name: Mendeley Data Data identification number: DOI: 10.17632/7rzcs2czxb.2 https://data.mendeley.com/datasets/7rzcs2czxb/2

## Value of the Data

The data collected provide information, from a gender perspective, about the characteristics of women in their personal, family, economic, and financial life, as well as their leadership and public participation roles. These data provide information on the ability of women to make decisions, based on their perception and action, concerning their lives in the dimensions mentioned above, which is of great value to the universe of statistical data for the region and the country.

The database is of interest to researchers who wish to make comparisons with other national and international statistical data on gender and intersectional issues. Specifically, this data set is comparable with the statistical production of gender issues in countries such as Mexico, Peru, and Argentina, among others. From this data set, it is possible to estimate indicators of quality of life, political culture, time use, health, household composition, and financial inclusion, among others, all comparable to estimates, at the country level, of others National databases

Along the same line, it is worth adding that this set of data allows comparisons to be made also between subnational units. It is valuable to highlight the specificity of the conditions of the autonomy of women in the Colombian Pacific. As a result, this database allows quantitative approaches to identify the central variables or factors in the conditions of women's inequality. In other words, it allows designing and enriching, from a parsimonious perspective, theoretical models that address equity based on the realities of women in the Cauca Valley.

In general terms, the data allow the identification of correlations between the three dimensions of autonomy: personal and family; economic and financial; and leadership and public participation. It also determines the degree of interactions among dimensions. This makes our data set articulate variables that take into account women's practices and ways of conceiving the social realities in which they are involved.

## 1 Data Description

The data presented were collected between October 18 and November 27, 2018, through face-to-face surveys applied to women 18 years of age or older. The questionnaire was designed by the team of researchers and approved by academic peers^1^ with expertise in gender studies and quantitative analysis. Female respondents answered questions related to socio-demographic characterization, their living conditions, and their decision-making capacity, from symbolic and material aspects, in the following dimensions: personal-family, economic-financial, and leadership and public participation.

Our observation units were women of legal age, households, and geographic blocks. The sample was applied in four municipalities of Valle de Cauca: 1) Cali as the central municipality of the entire Colombian Pacific region, being the third-largest city in the country and the capital of Valle del Cauca; 2) Jamundí, which is a municipality near Cali that shares certain dynamics with it; 3) Buenaventura as the most important seaport in Colombia; and finally 4), Yumbo, configured as one of the main industrial cities of the Pacific region. We implemented a multi-stage probability sampling method to select units by simple random sampling. The sample was stratified by political division and socioeconomic strata. The political division is by Urban Planning Units (UPU) 2 in the case of Cali, and by comunas^3^ for Yumbo, Jamundi and Buenaventura. For socioeconomic levels, we proposed classification by public service provider companies; i.e., low socioeconomic level 1 and 2; medium socioeconomic level 3; high socioeconomic level 4, 5 and 6. Based on these two dimensions (political division and socioeconomic strata), we performed simple random sampling, first applied to geographic blocks, then to households, and finally, to a person within the household. For this last selection, we chose the woman in the household whose birthday was closest to the day of the survey. However, preference was given to transgender women if there were any in the household. Table 1 shows the number of surveys applied according to socioeconomic level and municipality.

**Table 1:** Frequency of instruments applied by socioeconomic level and municipality

Municipality	Socioeconomic level 1	Socioeconomic level 2	Socioeconomic level 3	Socioeconomic level 4	Socioeconomic level 5 y 6	Total
**Buenaventura**	183	88	117	13		401
**Cali**	132	234	262	105	79	812
**Jamundí**	26	73	44	4		147
**Yumbo**	56	62	29			147
**Total**	397	457	452	122	79	**1507**

The questionnaire contains 74 questions distributed as follows: 23 questions of sociodemographic characterization, 24 questions of physical autonomy, 16 of economic autonomy and 11 questions of leadership and public participation. Application time ranged from 60 to 90 minutes. Unprocessed data from the women's autonomy survey are attached as supplementary material. Finally, the general characteristics of the women surveyed are presented in Table 2.^4^

**Table 2:** General characteristics of the sample (1507 women). 2018

**Age range**	
18 - 24 (%)	7.44
25 - 34 (%)	15.14
35 - 44 (%)	17.40
45 - 54 (%)	17.60
55 - 64 (%)	18.46
Over 64 (%)	23.97
**Ethnic self-recognition**	
Afro-Colombian, Afro-descendant (black, mulatta, raizal, palenquera) (%)	39.11
Mestiza (%)	30.68
White (%)	18.06
None (%)	5.98
Indigenous (%)	4.32
Other (%)	1.86
**Sex registered at birth**	
Man (%)	0.60
Woman (%)	99.40
**Self-identification of gender**	
Woman (%)	99.40
Transgender woman (%)	0.60
**Educational level**	
High School (%)	26.03
High School Incomplete (IC) (%)	18.13
Technical or technological (%)	16.20
Primary IC (%)	12.15
Primary (%)	10.82
Undergraduate (%)	6.51
Graduate (%)	3.12
Undergraduate IC (%)	3.05
Technical or technological IC (%)	1.53
None (%)	1.33
Graduate IC (%)	1.13
**Current work situation**	
Housework (%)	27.29
Unemployed and looking for work (%)	20.58
Freelance or self-employed (%)	17.33
Employed in formal work (%)	10.56
Unemployed and not looking for work (%)	5.38
Retired (%)	4.18
Employed in informal work (%)	3.32
Studying (%)	2.72
Freelance professional (%)	2.46
Has never worked (%)	2.12
Catalogue saleswoman (%)	1.46
Employed and looking for work (%)	1.39
Own business with at least one employee (%)	0.66
Family business with at least one employee (%)	0.53
**Level of income**	
Between 1 and 2 legal monthly minimum wages (LMMW) (%)	13.41
Between 3 and Entre 4 LMMW (%)	1.53
More than 5 LMMW (%)	0.27
Less than 1 LMMW (%)	22.18
N/A (%)	62.28
Doesn't know (%)	0.33
**Marital status**	
Free union (%)	34.40
Single (%)	31.01
Married (%)	17.86
Widowed (%)	9.43
Separated/Divorced (%)	7.30

## 2 Experimental Design, Materials, and Methods

To deal with the challenges of data collection, the design of the measurement instrument required an exhaustive review of other national-level surveys that addressed the proposed dimensions of analysis to measure personal-family, economic-financial, and leadership and public participation autonomy (Departamento Administrativo Nacional de Estadística [DANE], [2,3,6]. Although disaggregated data were presented for Colombia, there was no instrument to articulate the proposed dimensions. Likewise, data that characterized these dimensions with a gender perspective were scarce. Based on the foregoing, we put forward the present measurement proposal, specifically for women, articulating -as much as possible- the national forms of measurement for future comparison exercises.

For the design of the instrument, the work team made up of the authors of this article, reviewed multiple measurement surveys in order to identify variables that can be compared at the national and regional level with the sub-national units that our measurement addresses. In addition, questions were designed to characterize the autonomy of women in Valle del Cauca, a purpose absen°t in the National Statistical System of Colombia.

In this sense, for the dimension of personal and family autonomy, specifically in the questions of sexual and reproductive rights, we take as reference some questions from the *Encuesta Nacional de Demografía y Salud* (2015*)*. Regarding the time use of the respondents, our reference was the *Encuesta Nacional de Uso del Tiempo* (2016). The violence section was referenced in the *Encuesta Dinámica de Relación en los Hogares de México* (2018) [5] with the purpose of expanding the battery of data on gender-based violence for Colombia. In this dimension, the team's own questions are aimed at women's decision-making, following the central objective of the survey: to characterize women's agency in these dimensions.

For the dimension of economic and financial autonomy, like the previous dimension, the questions of access to economic resources or characterization of the labor relationship are referenced from the *Encuesta Nacional de Demografía y Salud* (2015), *Encuesta Nacional de Uso del Tiempo* (2016) and the *Gran Encuesta de Hogares* (2006). Likewise, the questions that point to the level of decision and agency of women in this dimension are of own elaboration.

Lastly, for Leadership and Public Participation in terms of electoral and community participation the *Encuesta de Cultura Política* (2013) [4] was a reference and for the public space the report *Ella se mueve Segura* [1] of Argentina. In this dimension, the questions elaborated by the team account for the type of participation that women have had, their willingness to participate and the barriers to it.

For fieldwork, the OEM hired a national company for data collection. It is worth clarifying that although the interviewers were trained in this respect^5^, the first fieldwork reports identified shortcomings. These were related to the interviewers' lack of expertise in relation to the gender perspective, and their lack of expertise in knowing when they should continue with the list of questions and which questions they should omit. Another major challenge concerns the length of the survey, whereby given the large number of questions, there was a high risk of respondents becoming fatigued. Hence, the interviewers were forced to adopt in situ strategies to be able to complete the form. Where this was not possible, interviewees were contacted by phone. During fieldwork, a high proportion of women who did not participate in the labor market and older adults (over 65 years) were found during the fieldwork, because age and non-employment meant that these women spend more time at home. Based on this information, an additional application session was scheduled for after 6:00 p.m.^6^ and weekends as a strategy to reach women with formal employment.

Contextual difficulties included public order problems in Buenaventura, which meant that we had to postpone visits to comunas 11 and 12 of that municipality until further notice. The application schedule for these comunas was only until 3:00 p.m. In Cali, the highest socioeconomic levels (5 and 6) resisted engaging with the interviewers, which implied scheduling appointments by telephone. At this point, the OEM's relationship with Fundación WWB Colombia and Universidad Icesi facilitated the scheduling of appointments. In sum, 1,507 women were surveyed in Cali, Jamundí, Buenaventura, and Yumbo, and the data collected are being used to generate recommendations for the formulation of public policies in Valle del Cauca and the Colombian Pacific region.

## Declaration of Competing Interest

The authors declare that they have no known competing financial interests or personal relationships which have, or could be perceived to have, influenced the work reported in this article.

## Appendix Supplementary materials

Image, application 1

Image, application 2

Image, application 3

## Footnotes

1 The expert peers who reviewed the measurement instruments were: Carolina Vergel (Universidad Externado de Colombia), Claudia Rodríguez (Women´s Link World), Helena Alviar (Universidad de los Andes), Isabel Jaramillo (Universidad de los Andes), Javier Pineda (Universidad de los Andes), Juan Carlos Rivillas (Profamilia), Laura Porras (Universidad del Rosario), Lina Céspedes (Universidad del Rosario), Natalia Salas (Universidad Icesi), and Sary Levy (Fundación WWB Colombia).

2 This election was based on the transition from the municipality of Santiago de Cali to the Special District. In 2018, the UPUs were the political division projected for the municipality.

3 The *comunas* are the administrative units in some municipalities in Colombia through which the urban area of a middle or main municipality is divided, grouping neighborhoods or sectors.

4 The income was consulted in current legal monthly minimum wages (LMMW), which are equivalent to

5 The company was asked to hire only women as interviewers given the content of the instrument.

6 In Colombia, the working day oscillates between 8:00 a.m. and 6:00 p.m.
