# Supplementary material for: The conditions for women's autonomy: Statistical data for Valle del Cauca
Source: Data Brief. 2020 May 21;31:105751. doi: 10.1016/j.dib.2020.105751 (PMC7262404; doi:10.1016/j.dib.2020.105751)
Supplement: Supplementary file 3 [file mmc3.docx]

**MEASUREMENT OF AUTONOMY OF WOMEN OBSERVATORY FOR THE EQUITY OF WOMEN CALI - JAMUNDÍ - YUMBO – BUENAVENTURA**

| “Good morning. My name is ______ and I work for the Observatory for Women's Equity, of the ICESI University and the WWB Colombia Foundation. We are conducting a citywide survey of women over 18 on issues associated with family life, the economy and public participation. ”  (In the event that a person other than a woman over the age of 18 opens the door, request the presence of one who inhabits the house and meets this condition. Once it is presented, it is presented again and the following information is given)  "Good Morning. My name is ______ and I work for the National Consulting Center, we are conducting a study for the Observatory for the Equity of Women, of the ICESI University and the WWB Colombia Foundation. We are conducting a citywide survey of women over 18 on issues associated with family life, the economy and public participation. This information will be used only for statistical and academic purposes to influence policies in favor of women's equity. This according to Law 1581 of 2012 on the protection of personal data. All the information you provide will be kept strictly confidential and will not be shown to other people. The survey takes approximately 1 hour. Your participation in this interview is voluntary and if there is any question you would not like to answer let me know and I will continue with the following questions. We look forward to having you, since your participation is very important for this process. Do you agree?  For any concern you can contact the National Consulting Center, telephone (2) 6674226. | | |
| --- | --- | --- |
|  | **Initiation** Hour / ___ / ___ / minutes / ___ / ___ / |  |

| **Section 1: SOCIODEMOGRAPHICAL INFORMATION OF THE SURVEYOF BIRTH** |
| --- |

1. DATE

| D | D | M | M | Y | Y | Y | Y |
| --- | --- | --- | --- | --- | --- | --- | --- |

1. Depending on your culture, customs, or traditions, you identify yourself as **(E: Read options, SA)**

| Afro-Colombian, Afro-descendant (black, mulatto, root, palenquera) | 1 |
| --- | --- |
| Indigenous | 2 |
| Mestizo | 3 |
| White | 4 |
| Rom or gypsy | 5 |
| Other | 7 |
| None | 8 |

1. Record sex at birth **(E: Read options, SA)**

| Woman | 1 |
| --- | --- |
| Man | 2 |

1. Do you identify yourself as? **(I: Read options, SA, give the clarification definition if the respondent asks you for the meaning of the term)**

| Woman | 01 | Continue |
| --- | --- | --- |
| Transgender woman (you were registered as a man but identify yourself as a woman) | 02 | Continue |
| Man | 03 | Thank and finish |
| Other. Which one? _________________ |  | Continue |

1. Do you agree to answer your sexual orientation?

| Yes | 1 | Continue |
| --- | --- | --- |
| No | 2 | Go to Q7 |

1. What is your sexual orientation? **(I: Read options, SA, * Give the clarification definition if the respondent asks you what sexual orientation is: It corresponds to your emotional erotic attraction for another person)**

| Heterosexual (for people of the opposite sex to yours, in this case men) | 01 |
| --- | --- |
| Homosexual (for people of the same sex of yours, in this case women) | 02 |
| Bisexual (for people of the opposite sex and of the same sex of yours, in this case men and women) | 03 |
| Other _ Which one? ____ |  |

1. What is the highest educational level attained by you? **(E: Read options, SA) (Show card P8)**

| Educational Level | cod | Instruction | Educational Level | cod | Instruction |
| --- | --- | --- | --- | --- | --- |
| None | 01 | Continue | Undergraduate incomplete | 09 | Continue |
| Primary complete | 02 | Pass to 10 | Complete specialization | 10 | Pass to 10 |
| Primary incomplete | 03 | Continue | Specialization incomplete | 11 | Continue |
| Complete high school | 04 | Pass to 10 | Full Masters | 12 | Pass to 10 |
| Incomplete Baccalaureate | 05 | Continue | Incomplete Master's Degree | 13 | Continue |
| Full Technological or Technological | 06 | Pass to 10 | Full Doctorate | 14 | Go to 10 |
| Technician or technician incomplete | 07 | Continue | Doctorate incomplete | 15 | Continue |
| Undergraduate complete | 08 | Go to 10 |  |  |  |

8A. Up to what level / grade / semester?

1. Why did you not do or suspend your studies? **(E: Read options, MA) (show card P8)**

| For economic reasons | 01 |
| --- | --- |
| For dedicating to household chores | 02 |
| Because you became pregnant | 03 |
| For dedicating yourself to the care of children | 04 |
| For dedicating yourself to the care of parents or relatives | 05 |
| For dedicating yourself to work and earn money | 06 |
| Because he married or went to live with his partner | 07 |
| Because he did not want to study anymore | 08 |
| Because he was difficult to study | 09 |
| Because of the internal armed conflict | 10 |
| Because of displacement | 11 |
| Because of pressure from his partner or family | 12 He |
| is currently studying | 13 |
| Other __ What? ________ |  |

1. What is your current employment status? **(E: Read options, SA) (show card P9)**

| Unemployed and looking for work - | 01 | Go to P12 |
| --- | --- | --- |
| Unemployed and not looking for work | 02 | Go to P12 |
| Employee and looking for work | 03 | Continue |
| Employee with formal employment | 04 | Continue |
| Employee with informal employment | 05 | Continue |
| Professional Freelance | 06 | Continue |
| Freelance or self-employed worker | 07 | Continue |
| Own business with at least one employee | 08 | Continue |
| Family business with at least one employee | 09 | Continue |
| Housework (housewife working in household) | 10 | Go to P12 |
| Pensioned | 11 | Go to P12 |
| Saleswoman by catalog | 12 | Continue |
| Never worked | 13 | Go to P12 |
| You are studying | 14 | Go to P12 |

1. Income level: Before discounts (social security, withholding tax, among others), how much did you earn for your work? (include tips and commissions and exclude payments in kind) (**E: The answer is open and the pollster marks the closest ones)**

| Less than 1 SMLV (790,000 COP approx.) | 01 |
| --- | --- |
| Between 1 and 2 SMLV (Between 1,580,000 and 2,370,000 COP approx.) | 02 |
| Between 3 and 4 SMLV (Between 2,370,000 and 3,160,000 COP approx.) | 03 |
| Between 4 and 5 SMLV (Between 3,160,000 and 3,950,000 COP approx.) | 04 |
| More than 5 SMLV (More than 3,950,000 COP) | 05 |

1. How much do your monthly income add up for other items? (child support, remittances, leases, among others) **(E: This question is asked of all women, INCLUDING THOSE WHO ARE NOT EMPLOYED)**
2. What is their marital status? **(E: Read options, SA)**

| Single | 01 |
| --- | --- |
| Free union | 02 |
| Married | 03 |
| Separated / Divorced | 04 |
| Widow | 05 |
| Other __ Which one? ______ |  |

1. As for your current partner situation, you… **(E: Read options, SA) You**

| have a partner with whom you cohabit (Live together) | 1 | Continue You |
| --- | --- | --- |
| have a partner with whom you do not cohabit (it is a couple that shares family expenses and make family decisions with you) | 2 | Continue |
| Have more than one partner | 3 | Continue |
| Do not have a partner | 4 | Go to 16 |
| Have a partner with whom you do not cohabit and **do not** share family expenses or make family decisions with you | 5 | Go to 16 |

1. How long ago they are a couple? **(E: Main couple in case of more than one)**

| Years | | Months | |
| --- | --- | --- | --- |
|  |  |  |  |

1. How many people depend economically on you?

|  |  |
| --- | --- |

**The following questions are related to your home and your home**

1. Who composes the home in which you live?  **(E: Read options, SA)**

| Only you | 1 |
| --- | --- |
| You and your son (s) or daughter (s) | 2 |
| You, your partner and your children (s) or daughter (s) | 3 |
| You with your partner | 4 |
| You, your partner, your children and other relatives | 5 |
| You and other relatives other than children and partner | 6 |
| You and other non-relatives | 7 |
| You, your children and other relatives | 8 |
| Other, which one? |  |

1. According to the receipt of electricity and public services, what is the stratum of your residence?

|  |  |
| --- | --- |

1. The dwelling that currently lives is… **(E: Read options, SA)**

| Family | 1 |
| --- | --- |
| Own with mortgage | 2 |
| Own canceled | 3 |
| Rented | 4 |
| Sublet (share rent) | 5 |
| Housing assigned by the State, for example step | 6 home |
| In the place where it is used | 7 |
| Another, which one? |  |

1. Which of the following public, private or communal services does this home have? **(E: Read options, MA)**

| Electricity | 01 |
| --- | --- |
| Natural gas connected to public network | 02 |
| Aqueduct | 03 |
| Sewer | 04 |
| Garbage collection. | 05 |
| Internet | 06 |
| Cable television | 07 |
| None | 88 |

20A. Do you have water count? **(E: Answer this question if you marked 03 on P20)**

| Yes | 1 |
| --- | --- |
| No | 2 |

20B. How many times per week do you pick up the trash? (**E: Answer this question if you marked 05 on P20, if you don't know dial 99)**

|  |  |
| --- | --- |

1. This home has and uses **(E: Read options, MA)**

| Clothes washing machine | 01 |
| --- | --- |
| Fridge or refrigerator | 02 |
| Blender | 03 |
| Iron | 04 |
| Electric or gas | 05 |
| stoveOven electric, gas or microwave oven | 06 |
| Dishwashing machine | 07 |
| Television | 08 |
| Sound or radio equipment | 09 |
| Motorbike (working by someone other than someone in your home) | 10 |
| Bicycle | 11 |
| Landline | 12 |
| Private car | 13 |
| Private motorcycle | 14 |

21A. The bicycle is used as… **(E: Answer this question if you marked 11 on P21)**

| As usual means of transportation | 1 |
| --- | --- |
| Recreational or sports use | 2 |

1. Does this household have a domestic service contracted ?: Domestic employee, babysitter (a), gardener, nurse, butler, housekeeper, among others

| Yes | 1 |
| --- | --- |
| No | 2 |

1. The space of the dwelling in which you stay longer is **(E: Read options, SA)**

| Kitchen | 01 |
| --- | --- |
| Dining / dining room | 02 |
| Studying room | 03 |
| Balcony or hallway | 04 |
| Work space or business inside the house | 05  06 |
| Own room or shared with the couple / children |  |
| Garden or patio | 07 |
| Place for laundry | 08 |
| TV room | 09 |
| The whole house does not have a specific space | 10 |
| Traveling | 11 |
| Other __ Which one? ___ |  |

1. The main reason for using this space more than others is **(E: Read options, SA)**

| My responsibilities with domestic work | 1 |
| --- | --- |
| My responsibilities with caring for other people (children, children, sick people, elderly people). | 2 |
| My rest and recreation | 3 |
| A productive activity (sewing, attending a store, working in a workshop). | 4 |
| Share time with the family | 5 |
| Study | 6 |
| Other, which |  |

**Section 2: USE OF THE SURVEY TIME**

**The following questions ask about how you use and distribute your time spend**

1. In what activity did you most of your time last week ?: **(E : Read options, SA)**

| Unpaid care work | 01 |
| --- | --- |
| Looking for work | 02 |
| In personal, medical and leisure activities | 03 |
| Working or studying | 04 |
| None | 88 |

24.B       Please indicate, on average of one day a day (24 hours), how do you distribute your time? **(E: Read all the answer options and indicate the hours HH and minutes MM for each activity. If it does not apply, write 0 (zero))**

|  | **Hours** | | **Minutes** | |
| --- | --- | --- | --- | --- |
| 24b.1 Employment outside your home |  |  |  |  |
| 24b.2 Work in your own company or business |  |  |  |  |
| 24b.3 Domestic work |  |  |  |  |
| 24b.4 Unpaid care of family members or others |  |  |  |  |
| 24b.5 study |  |  |  |  |
| 24b.6 Exercise or sport |  |  |  |  |
| 24b.7 Dream |  |  |  |  |
| 24b.8 Leisure activities with your family |  |  |  |  |
| 24b.9 Personal activities alone |  |  |  |  |
| 24b.10 Displacement |  |  |  |  |
| Other __ Which one? ___ |  |  |  |  |

25. In your home, who is the person who mostly assumes the following tasks? **(I: Read all tasks and mark the option indicated by the respondent for each one (only one option per task)) We are talking about who executes the activity Show card P25**

|  | You | Your partner | You and your partner | A family member | A third party | You and a family member | N / A |
| --- | --- | --- | --- | --- | --- | --- | --- |
| 25.1 Buy food, medicines and household goods | 1 | 2 | 3 | 4 | 5 | 6 | 88 |
| 25.2 Cook or supply food to household members | 1 | 2 | 3 | 4 | 5 | 6 | 88 |
| 25.3Wash, iron, sew or organize the clothes or shoes of household members | 1 | 2 | 3 | 4 | 5 | 6 | 88 |
| 25.4Clean this house (mop, sweep ...) | 1 | 2 | 3 | 4 | 5 | 6 | 88 |
| 25.5Clean the vehicle of the home | 1 | 2 | 3 | 4 | 5 | 6 | 88 |
| 25.6Caring for pets or the garden | 1 | 2 | 3 | 4 | 5 | 6 | 88 |
| 25.7Bring water or fuels for the operation of the home | 1 | 2 | 3 | 4 | 5 | 6 | 88 |
| 25.8Go to pay bills or do household paperwork | 1 | 2 | 3 | 4 | 5 | 6 | 88 |
| 25.9Direct or coordinate activities of domestic workers | 1 | 2 | 3 | 4 | 5 | 6 | 88 |
| 25.10Caring for people under 5 | 1 | 2 | 3 | 4 | 5 | 6 | 88 |
| 25.11Caring for people over 60 | 1 | 2 | 3 | 4 | 5 | 6 | 88 |
| 25.12Caring for sick or disabled people | 1 | 2 | 3 | 4 | 5 | 6 | 88 |
| 25.13Accompany medical / dental appointments / exams | 1 | 2 | 3 | 4 | 5 | 6 | 88 |
| 25.14 Advise or comfort a household member | 1 | 2 | 3 | 4 | 5 | 6 | 88 |
| 25.15 Take or tr Somewhere in which the person plays sports, entertains or educates | 1 | 2 | 3 | 4 | 5 | 6 | 88 |
| 25.16 Play, read or other recreational activities for others | 1 | 2 | 3 | 4 | 5 | 6 | 88 |
| 25.17 Dress, clean and feed a household member | 1 | 2 | 3 | 4 | 5 | 6 | 88 |
| 25.18 Support others in school activities | 1 | 2 | 3 | 4 | 5 | 6 | 88 |

1. What are the different places where you stay and work where you stay the longest?

| 0 | Does not leave |
| --- | --- |
| 1 | Places that respond to work responsibilities / domestic work or study: Activities of children, client house or where you work, place of study. |
| 2 | Other houses of close people: House of friends, house of the couple, house of relatives. |
| 3 | Daily places: City center, commuting, in the street in general, medical services, supermarket |
| 4 | Leisure and other activities: Shopping center, recreational center, cinema, gym, paruqes and green areas, going out with friends, church. |

1. How old were you when your first pregnancy occurred?

| 1 | Over 19 years |
| --- | --- |
| 0 | Under 19 |
| 88 | Does not apply |

1. Do you freely make decisions about the exercise of your sexual orientation?

| 0 | Never |
| --- | --- |
| 1 | Almost Never |
| 3 | Sometimes |
| 4 | Almost Always |
| 5 | Always |

1. Do you freely make decisions about the choice of your erotic-affective partners? (Erotic affective: people with whom you have romantic and / or sexual relations)

| 0 | Never |
| --- | --- |
| 1 | Almost Never |
| 3 | Sometimes |
| 4 | Almost Always |
| 5 | Always |

1. Do you freely make decisions about your sexual practices?

| 0 | Never |
| --- | --- |
| 1 | Almost Never |
| 3 | Sometimes |
| 4 | Almost Always |
| 5 | Always |

1. In his daily activities he presents permanent difficulties for **(E: Read options, RM) (SAMPLE CARD P31)**

| Hear the voice or the sounds | 01 |
| --- | --- |
| Talk or talk | 02 |
| See close, far or around | 03 |
| Move the body, walk, climb or lower Stairs | 04 |
| Grab or move objects with your hands | 05 |
| Eat, dress or bathe on your own | 06 |
| Understand, remember or make decisions for yourself | 07 |
| Relate or interact with other people | 08 |
| Develop in your daily life because of some mental health situation | 09 |
| Do daily tasks without showing heart, respiratory or kidney problems | 10 |
| Mobilize, so you should use a wheelchair | 11 |
| None | 88 |

1. Do you have friendships with which you have interaction? (I: if you don't understand the question, say: Do you have friends with whom, despite not seeing yourself permanently interact)?

| Yes | 1 | Continue |
| --- | --- | --- |
| No | 2 | Go to P66 |

1. In general, how are the decisions associated with the distribution of domestic work and home care taken?**(I: Read options, SA)**

| You take them | 1 |
| --- | --- |
| They take your partner | 2 |
| They take them both | 3 |
| They take the household members considering the time available | 4 It |
| takes another person | 5 |

1. In your case, how much does the following statement apply? Working outside the home is a fundamental aspect that has allowed you to act freely and actively participate in family decisions **(E: Read options, SA)**

| Strongly agree | 1 |
| --- | --- |
| Agree | 2 |
| Disagree | 3 |
| Strongly disagree | 4 |
| Do not work outside your home | 5 |

**Section 7: VIOLENCE AGAINST WOMEN**

**(E: The interviewer should be patiently and calmly available for the following questions, avoid any type of facial, body or verbal expression that evidences some kind of bias or judgment about the respondents' responses. in case there is silence, give prudent time to respond. If you do not wish to answer any, remind the respondent that the answers are confidential and anonymous, without pressing them to do so. Ensure that these questions are answer without the presence of other people)**

The questions that follow are associated with interpersonal relationships, especially with your partner. I know that some of these questions are very personal, but I assure you that your answers are completely confidential and will be of great importance to understand some aspects of violence against women.

1. Has your partner (or your former partner) ever had or had any of these reactions **(E: Deliver card # 71) (MA)**

| He has stopped talking or ignored | 01 |
| --- | --- |
| He has been angry because he talks to another man | 02 He |
| has accused her If you are unfaithful | 03 |
| It has prevented you from meeting your friends | 04 |
| You have tried to limit contact with your family | 05 |
| You have insisted on knowing where you are all the time | 06 |
| You have not consulted important family decisions | 07 |
| You have been told "you are useless", "You are a brute" "You do nothing right" | 08 He |
| has threatened to leave with another woman | 09 He |
| has threatened to leave her | 10 He |
| screams | 11 He |
| has pushed or shaken | 12 He |
| has hit her with his hand | 13 He |
| has hit her with a Object | 14 He |
| has kicked or dragged | 15 He |
| has attacked with a knife, firearm or other weapon | 16 He |
| has tried to strangle or burn it | 17 He |
| has watched the way you spend money | 18 He |
| has threatened to take away your financial support | 19 |
| He has work or study prohibited | 20 |
| Spent the money that was needed for the house | 21 |
| It has taken over or taken away money or property (property, land) | 23 |
| It has physically forced you to have sex or sexual acts that you did not want | 24 |
| Hit or throw things | 25 |
| Hit or mistreat someone other than you, for example, your children | 26 |
| Restricts your access to goods such as money, food, television, internet, telephone, transportation, etc. | 27 |
| Silence under threat or manipulation forcing you not to discuss the situation with family, friends, authorities, or acquaintances | 30 |
| None of the above | 88 |

1. According to your experience, do you consider your home to be a safe place for you: free of violence such as those previously mentioned? **(E: Read options, SA)**

| Very frequently | 1 |
| --- | --- |
| Frequently | 2 |
| Sometimes | 3 |
| Almost never | 4 |
| Never | 5 |

1. Do you know what to do or what legal instances you can go to if you experience some kind of gender violence against women?

| Yes | 1 |
| --- | --- |
| No | 2 |

For each of the following phrases, indicate if you agree or disagree on a scale of: agree, neither agree nor disagree or disagree

| **(A: Agree, NAND: NI de agree or disagree, D: Disagree)** | **A** | **NAND** | **D** |
| --- | --- | --- | --- |
| 1. There are certain circumstances where women are justified to be mistreated | 1 | 2 | 3 |
| 1. In general, it is preferable that male children receive more training than female daughters | 1 | 2 | 3 |
| 1. Violence based on gender against women (because of their status as women), they are a serious violation of human rights | 1 | 2 | 3 |
| 1. Men cannot control themselves. Violence is simply part of its nature | 1 | 2 | 3 |
| 1. Most women who suffer gender-based violence are abused by people they know, often people they trust and love | 1 | 2 | 3 |
| 1. Women who experience gender-based violence cause abuse through inappropriate behavior | 1 | 2 | 3 |
| 1. Women have the right to say "no" if they do not want to have sex with their partner | 1 | 2 | 3 |
| 1. Women who continue with their partners after being beaten is because they like | 1 | 2 | 3 |
| 1. If a woman tries to please a man, he will lovemore and not hit | 1 | 2 | 3 |
| 1. the men of the house are the ones who decide the distribution of care and domestic work | 1 | 2 | 3 |
|  |  |  |  |

**Section 8: FINANCIAL AUTONOMY (for all respondents)**

The following questions are related to economic aspects, such as your work, your income, and your access to financial products.

1. Indicate which of the following income you have had during the last month **(E: Read options, MA)**

| Own income from work | 01 |
| --- | --- |
| Income in kind such as food, housing or other | 02 |
| Food allowance in money | 03 |
| Transportation assistance in money | 04 |
| Family allowance in money | 05 |
| Income from business additional to your work | 06 |
| Income from pension, retirement , pension replacement, disability or old age | 07 |
| Income for support of children under 18 years | 08 |
| Income from rental of houses, apartments, recreational farms, lots, vehicles, machinery and equipment | 09 |
| Income from receipt of remittances from abroad | 10 |
| Other __ Which? __ |  |
| None | 88 |

1. During the last 12 MONTHS you received **(E: Read options, MA)**

| Premium of services, Christmas, holidays, bonuses or compensation | 01 |
| --- | --- |
| Income for aid in money from other homes or institutions | 02 |
| Income for sale of properties (houses, buildings, lots, machinery, vehicles, appliances, etc.) | 03 |
| Income from the sale of household appliances, furniture, books or household goods, among others | 04 |
| Other __ Which one? __ |  |
| None | 88 |

1. Who decides (decided) mainly how the money you earn (spent) was spent (spent) **(E: Read options, SA)**

| You | 1 |
| --- | --- |
| Your partner | 2 |
| Both | 3 |
| Someone else | 4 |
| Together with someone else | 5 |

1. Which ( es) of the following assets is your property and is it registered or titled in your name, either with your partner or with someone else? **(E: Read options, MA)**

| A residential property (house, apartment, etc.) | 01 |
| --- | --- |
| A vehicle (motorcycle or automobile) for personal use | 02 |
| A vehicle (taxi or car) for commercial purposes | 03 |
| A land or lot | 04 |
| A commercial premises | 05 |
| Animals such as cattle, chickens, pigs | 06 |
| None | 88 |

1. If I had an emergency today that required an expenditure close to 1 minimum wage (790 thousand pesos), how would I get most of the money? **(E: Read options, SA)**

| Your savings | 01 |
| --- | --- |
| Sale or commitment of any of your belongings | 02 |
| An advance of your salary | 03 |
| Covered by your partner | 04 |
| Loan from relatives, friends or acquaintances | 05 |
| Credit card or credit of financial entity | 06 |
| Loan to natural person (drop-drop, lender) | 07 |
| Other__ Which? |  |

1. In your home, most of the time, who decides how daily expenses are paid (rent, food services)? **(E: Read options, SA)**

| You | 01 |
| --- | --- |
| Your partner | 02 |
| You and your partner | 03 |
| Any of your parents | 04 |
| You in conjunction with someone else | 05 |
| Other (family, friend) |  |

1. In your home, how is payment of expenses organized? everyday? **(E: Read options, SA)**

| Through a common stock exchange (combining contributions from all members) | 01 |
| --- | --- |
| Through individual contributions (each contributor takes care of a specific expense) | 02 |
| You take care of all those expenses | 03 |
| Your partner takes care of all those expenses | 04 |
| You and your partner take care of these expenses | 05 |
| Another member of the household takes care of everything | 06 |
| There is no fixed scheme | 07 |
| Other Which one? |  |

1. In your home, most of the time, who decides how economic resources are managed when buying goods such as real estate or vehicles? **(E: Read options, SA)**

| You | 01 |
| --- | --- |
| Your partner | 02 |
| You and your partner | 03 |
| Any of your parents | 04 |
| You in conjunction with someone else | 05 |
| Other (family member, friend) |  |
| Not applicable |  |

1. In your home, how is the payment of daily expenses? (E: Read options, **SA**)

| Through a common stock exchange (combining contributions from all members) | 01 |
| --- | --- |
| Through individual contributions (each contributor takes care of a specific expense) | 02 |
| You take care of all those expenses | 03 |
| Your partner takes care of all those expenses | 04 |
| You and your partner take care of these expenses | 05 |
| Another member of the household takes care of everything | 06 |
| There is no fixed scheme | 07 |
| Other Which one? |  |

1. In your home, most of the time, who decides how economic resources are managed when buying goods such as real estate or vehicles? (E: Read options, **SA**)

| You | 01 |
| --- | --- |
| Your partner | 02 |
| You and your partner | 03 |
| Any of your parents | 04 |
| You together with someone else | 05 |
| Other (family member, friend) |  |
| Not applicable |  |

1. Indicate all the ways you save (E: Read options , RM)

| Savings account (bank-cooperative-association) | 01 | Continue |
| --- | --- | --- |
| Certificates fixed-term deposit (CDT) | 02 | Continue |
| Chains | 03 | Continue |
| Personal savings at home | 04 | Continue |
| Savings on funds and other groups | 05 | Continue |
| You have invested in goods or merchandise | 06 | Continue |
| You have lent money to receive some additional profit | 07 | Continue |
| None, do not save | 08 | Go to 103 |
| Other Which one? |  | Continue |

1. What approximate amount of your income goes to paying debts? **(E: Read options, SA)**

| Less than a quarter | 1 |
| --- | --- |
| Less than half | 2 |
| Half | 3 |
| More than half | 4 |
| Does not have a fixed amount | 5 |

1. Which of the following insurance do you currently have? **(E: Read options, MA)**

| Life | 01 |
| --- | --- |
| insuranceMortgage insurance | 02 |
| Exquial insurance | 03 |
| Personal accident insurance | 04 |
| SOATPension | 05 |
| insuranceinsurance | 06 |
| Occupational risk(ARL) | 07 |
| Microinsurance (home, funeral, business, family) | 08 |
| None of the previous | 88 |
| Don't know | 99 |
| Other, which one? |  |

1. Which of the following statements do you most agree with? **(E: Read options, SA) SHOW CARD P61**

| Before buying something carefully consider whether you can afford it | 1 He |
| --- | --- |
| prefers to live a day and does not worry about tomorrow. | 2 He |
| prefers to spend money than to save for the future. | 3 |
| Pay your bills on time. | 4 |
| You are willing to risk some of your own money when you make an investment. | 5 |
| Personally monitor your financial issues. | 6 |
| Make a budget of your expenses and strive to meet it | 7 |
| Set long-term financial goals and strive to achieve them. | 8 |
| The money is there to be spent. | 9 |

1. Would you like to work on your own or as a microentrepreneur?

| Yes | 1 | **Continue** |
| --- | --- | --- |
| No | 2 | **Go to NOTE A You** |
| already have your own business | 3 | **Go to 128** |
|  |  |  |

1. How many jobs do you have?

**Section 9: LEADERSHIP AND PUBLIC PARTICIPATION**

**The following questions are part of the last section of the survey and are associated with their participation and perceptions of public life, as well as their relationship with the public space.**

|  | Yes | No |
| --- | --- | --- |
| 1. Do you have or have had a public office of popular choice? | 1 | 2 |

1. In which of the following organizations and / or groups have you participated in the last year? **(E: read options, RM) (DELIVER CARD P65)**

| Churches, organizations and / or religious groups. . | 01 | Continue |
| --- | --- | --- |
| Community action meetings and other community action organizations. | 02 | Continue |
| Collectives that promote the rights of ethnic groups. | 03 | Continue |
| Collectives that promote women's rights. | 04 | Continue |
| Collectives that promote the rights of children and adolescents. | 05 | Continue |
| Collectives that promote the rights of victims of armed conflict | 06 | Continue |
| Associations, groups, clubs and / or recreational, sports, artistic and / or cultural groups. | 07 | Continue |
| Groups, groups, associations and / or environmental organizations. | 08 | Continue |
| Consumer associations | 09 | Continue |
| Political parties and / or movements. | 10 | Continue |
| Unions. | 11 | Continue |
| Other organization Which one? |  | Continue |
| You have not participated in any | 88 | Go to 145 |

1. What kind of participation have you had? **(E: Read option, check maximum 2)**

| Leadership | 01 |
| --- | --- |
| Founder | 02 |
| Participated in decisions with voice and vote | 03 |
| Participated as assistant | 04 |
| Participated in the execution of specific activities or tasks | 05 |
| Other Which one? |  |

1. To solve some kind of problem that affects you and / or your community, in the last year: **(I: Read options, MA)**

| Have you processed complaints, claims, requests and / or requests before the corresponding authorities? | 01 | Continue |
| --- | --- | --- |
| Have you resorted to media such as television, radio, internet and written press? | 02 | Continue |
| Have you asked any kind of civic leader or political leader for help? | 03 | Continue |
| Have you participated in protests, demonstrations and / or public marches? | 04 | Continue |
| Have you called meetings and collective work with members of your community? | 05 | Continue |
| Other Which one? |  | Continue |
| None | 88 | Go to 148 |

1. When there are elections, you… **(E: Read options, SA)**

| Always vote | 1 | Go to 150 |
| --- | --- | --- |
| Sometimes vote | 2 | Go to 150 |
| Never vote. | 3 | Continue |

1. When you have the opportunity to participate in politics, you: **(E: Read options, SA)**

| Postulate your name to be elected to a position of popular election | 01 | Go to P154 |
| --- | --- | --- |
| Promote actions for other women to be elected | 02 | Go to P154 |
| Promote Actions for men to be elected | 03 | Go to P154 |
| Consider that with your vote is enough | 04 | Go to P154 |
| You have not had the opportunity to participate | 05 | Go to P154 |
| Do not participate in any way | 06 | Continue |

**Section 10: PUBLIC SPACE**

Questions you will find below they relate to your perceptions and feelings regarding the public space, in terms of whether it represents a context in which you can feel your integrity safe and preserved.

1. In which of the following sites do you feel insecure? **(E: Read options, RM) (SHOW CARD P70)**

| The streets of your neighborhood. | 01 | Continue |
| --- | --- | --- |
| In the squares and parks near your residence. | 02 | Continue |
| In the city center. | 03 | Continue |
| On public transport. | 04 | Continue |
| On your job site. | 05 | Continue |
| In your study site | 06 | Continue |
| In the night establishments | 07 | Continue |
| In the rural areas of your municipality | 08 | Continue |
| The establishments providing health services | 09 | Continue |
| In recreation spaces (parks, swimming pools, discos, shopping centers, etc. ) | 10 | Continue |
| On streets in neighborhoods other than yours | 11 | Continue |
| Other __ Which one? __ |  | Continue |
| None | 88 | Go to P157 |

1. Which of the following actions do you take to resolve the feeling of insecurity? **(I: Read options, MA)**

| You don't leave your home. | 01 |
| --- | --- |
| Only leaves when your partner accompanies you. | 02 He |
| leaves his house with people other than his partner. | 03 |
| Depart by private transport, taxi or uber to the meeting place | 04 |
| Consult social networks (Facebook groups, mass messages of WhatAspp, among others) | 05 |
| Other __ Which one? __ |  |
| None | 88 |

1. In the last 3 months, you have heard or seen situations around your home such as… **(E: Read MA options)**

| Vandalism in homes or businesses | 1 |
| --- | --- |
| Alcohol consumption in the streets | 2 |
| Robberies or robberies | 3 |
| Violent bands or gangs | 4 |
| Sale or consumption of drugs | 5 |
| Frequent shooting with weapons | 6 |
| Other, which one? |  |
| None | 7 |

1. During the last 3 months, for fear of suffering a crime (robbery, sexual assault, kidnapping, among others), you changed your habits regarding **(E: Read options, MA)**

| Carrying things of value | 01 |
| --- | --- |
| Walking around your home, at night | 02 |
| Visit relatives or friends | 03 |
| Allow your children to leave your home | 04 |
| Use of some means of transport (bus, MIO, motorcycle, etc.) | 05 |
| Take a trip alone (national / international) | 06 |
| Attend parties or events | 07 |
| Other __ Which one? ___ |  |
| None | 88 |

1. Because of the armed conflict, have you been forced to change your place of residence, leave your belongings, vacate your home or separate from your family?

| Yes | 1 |
| --- | --- |
| No | 2 |

Observations

|  |
| --- |
